# Comparative study regarding the stability of a proximal ulnar stump with or without distal oblique bundle reconstruction during the Sauvé‒Kapandji procedure: a finite-element analysis

**DOI:** 10.3389/fbioe.2024.1482747

**Published:** 2025-01-06

**Authors:** Shanqing Yin, Chenxi Zhang, Yaopeng Huang, Jiadong Pan, Xin Wang, Xiaodong Liu

**Affiliations:** ^1^ Department of Orthopedics, Yangpu Hospital, School of Medicine, Tongji University, Shanghai, China; ^2^ Department of Plastic Reconstructive Surgery and Hand Microsurgery, Ningbo No. 6 Hospital, Ningbo, China

**Keywords:** finite-element analysis, modeling, distal oblique bundle, Sauve-Kapandji, proximal ulnar stump

## Abstract

**Background:**

The most common postoperative complication of the Sauvé‒Kapandji (S-K) procedure is proximal ulnar stump instability. The distal oblique bundle (DOB) is a stable tissue used to stabilize the distal radioulnar joint. This study created finite-element models of the distal oblique bundle (DOB) to determine its effect on the proximal ulnar stump instability encountered during the Sauvé‒Kapandji procedure.

**Purpose:**

We hypothesized that a proximal ulnar stump with distal oblique bundle reconstruction would provide greater stability than a proximal ulnar stump without distal oblique bundle reconstruction.

**Methods:**

Detailed CT imaging data acquired from a pathological specimen of the wrist joint were imported into a finite-element analysis software package, and the regions of interest, including bone, cartilage, ligaments and tendons, were extracted to create a 3-dimensional model. The volar/dorsal and medial/lateral displacements of the proximal ulnar stump and the stress changes exhibited by the bone and distal oblique bundle tendon were measured with and without DOB reconstruction under 60° pronation, neutrality, and 60° supination.

**Results:**

When utilizing DOB reconstruction, the displacement of the radius relative to the proximal ulna stump was approximately 17.89 mm in the neutral position. The bone stress values corresponding to the neutral position, 60° pronation and 60° pronation were 1.01, 18.32 and 14.69 MPa, respectively. The stress peaks of the DOB tendon structure corresponding to the neutral position, 60° pronation and 60° pronation were 0.07 MPa, 2.21 and 1.55 MPa, respectively. Without DOB reconstruction, the displacement of the radius relative to the proximal ulna stump was approximately 18.05 mm in the neutral position. Under 60° pronation and 60° supination, the displacement values were approximately 14.62 mm and 16.89 mm, respectively. The peak bone stress values corresponding to the neutral position, 60° pronation and 60° supination were 1.02, 18.29 MPa and 14.41 MPa, respectively. The stress peaks of the tendon structure corresponding to the neutral position, 60° pronation and 60° pronation were 0.03, 0.87 and 0.85 MPa, respectively.

**Conclusion:**

DOB reconstruction is capable of improving the stability of the proximal ulnar stump during the Sauvé–Kapandji procedure.

## 1 Introduction

The Sauvé‒Kapandji (S-K) procedure is a commonly used procedure for the treatment of distal radioulnar arthritis (Minami et al., 2005), and the instability of the proximal ulnar stump is the most common complication that can occur after the SK procedure, which may cause severe pain during forearm rotation (Verhiel et al., 2019). At present, the extensor carpi ulnaris (ECU) is most commonly used to fix the proximal ulnar stump and increase its stability (Minami et al., 2006). However, the ECU is a dynamic tissue, and its stabilizing power may be insufficient (Kim et al., 2021). Jongmin Kim et al. (Kim et al., 2021) suggested combining the ECU tendon with an interosseous membrane (a combination of dynamic and static structures) to fix the proximal ulnar stump.

The DOB is the thickest part of the interosseous membrane (DIOM) of the distal forearm, and its existence rate is approximately 40% (Angelis et al., 2019a). It has been reported that (Angelis et al., 2019b) the DOB is a strong static structure that stabilizes the distal radioulnar joint (DRUJ). Hisao Moritomo et al. (Moritomo, 2012) hypothesized that the DOB also plays an important role in maintaining the stability of the proximal ulnar stump after performing the Sauve Kapandji procedure. However, few controlled studies have confirmed this theory. Therefore, we created finite-element models to compare the stability levels of the proximal ulnar stump with and without the DOB during the Sauvé–Kapandji procedure.

## 2 Methods

### 2.1 Anatomically based model geometry

The basic workflow of CT-based 3D wrist joint reconstruction involved several steps. First, CT image data concerning the wrist joint were obtained from a hospital. A DICOM file consisting of wrist joint CT scan data was selected from each voluntary participant, whose scans were taken at standard positions (including frontal, lateral, and dynamic views), ensuring that exclusion criteria such as arm deformities, tumors, infections, and prior surgeries on the arm or wrist joint were satisfied, thereby obtaining wrist joint image data.

**FIGURE 1:**
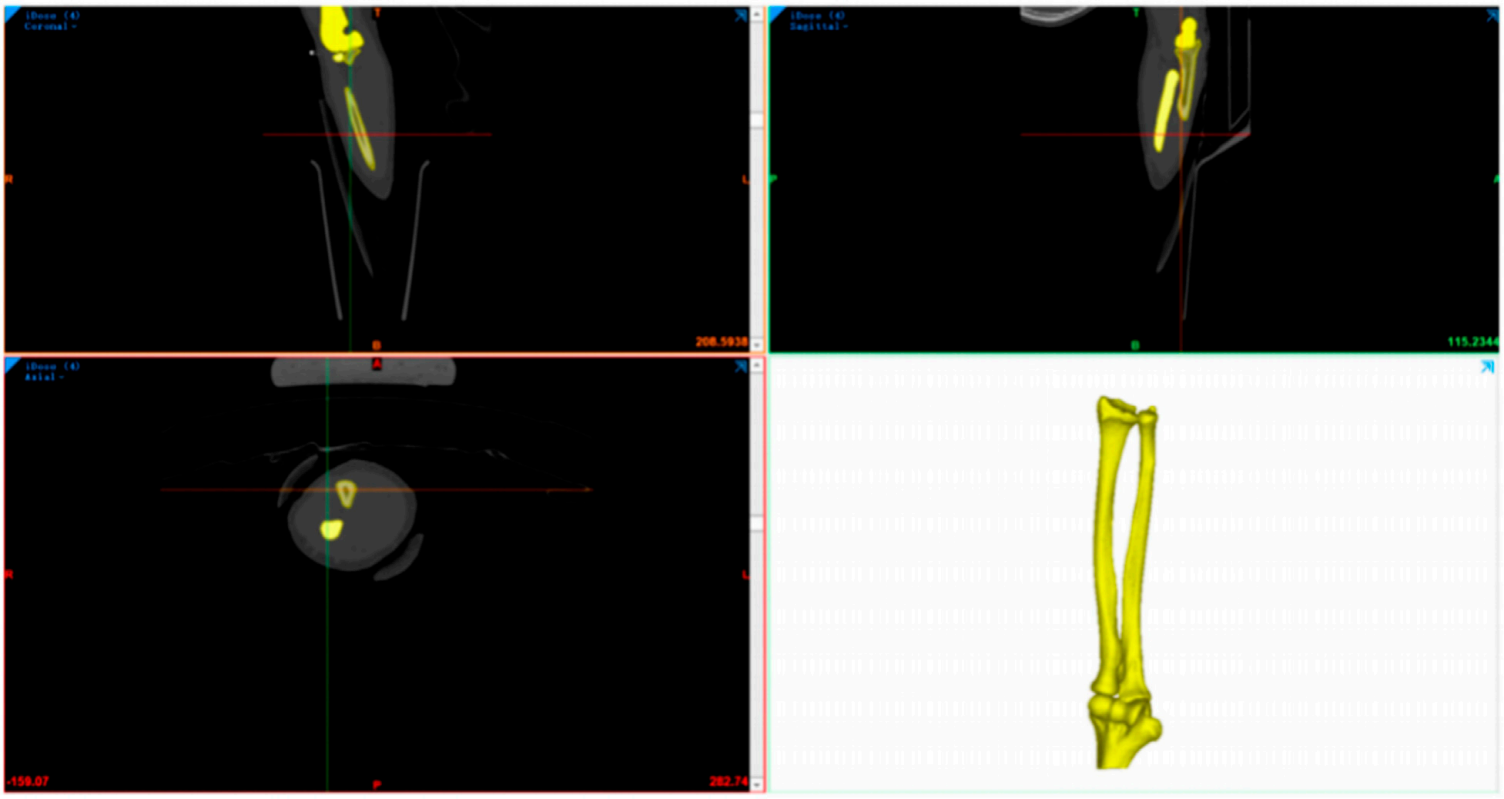
Three–dimensional reconstruction bone map of the CT wrist joint..

Next, Mimics 21.0 software (Materialise Company, Belgium) was used for data extraction to reconstruct the wrist joint (including the radius, ulna, humerus, etc.) as an STL model (Figure 1).

It was further processed with the subsequent finite-element preprocessing step implemented via MSC. Patran 2019 software (NASA Company, America), and finite-element postprocessing was performed using MSC. Nastran 2019 software (NASA Company, America) to set finite-element mesh properties, define the material parameters, apply loads, implement boundary condition constraints, and conduct analyses in various computational scenarios.

#### 2.1.1 Image processing stage

To convert 2D CT data into a 3D solid structure, DICOM files need to undergo conversion and processing. The image data reconstruction procedure primarily consisted of four parts:Image Input Phase; hreshold Determination Phase; Segmentation, Image Filling, and 3D Model Generation; Optimization of the 3D Geometry Model.

#### 2.1.2 Surface fitting and solid geometric model processing

The geometric model of the wrist joint structure obtained from the 3D CT reconstruction process was initially rough and consisted of triangular mesh models with deformities, distortions, and overly rough surfaces. To address these issues, Through precise surface processes, it achieves surface smoothing and ultimately forms a three-dimensional geometric model of the wrist joint.

**FIGURE 2:**
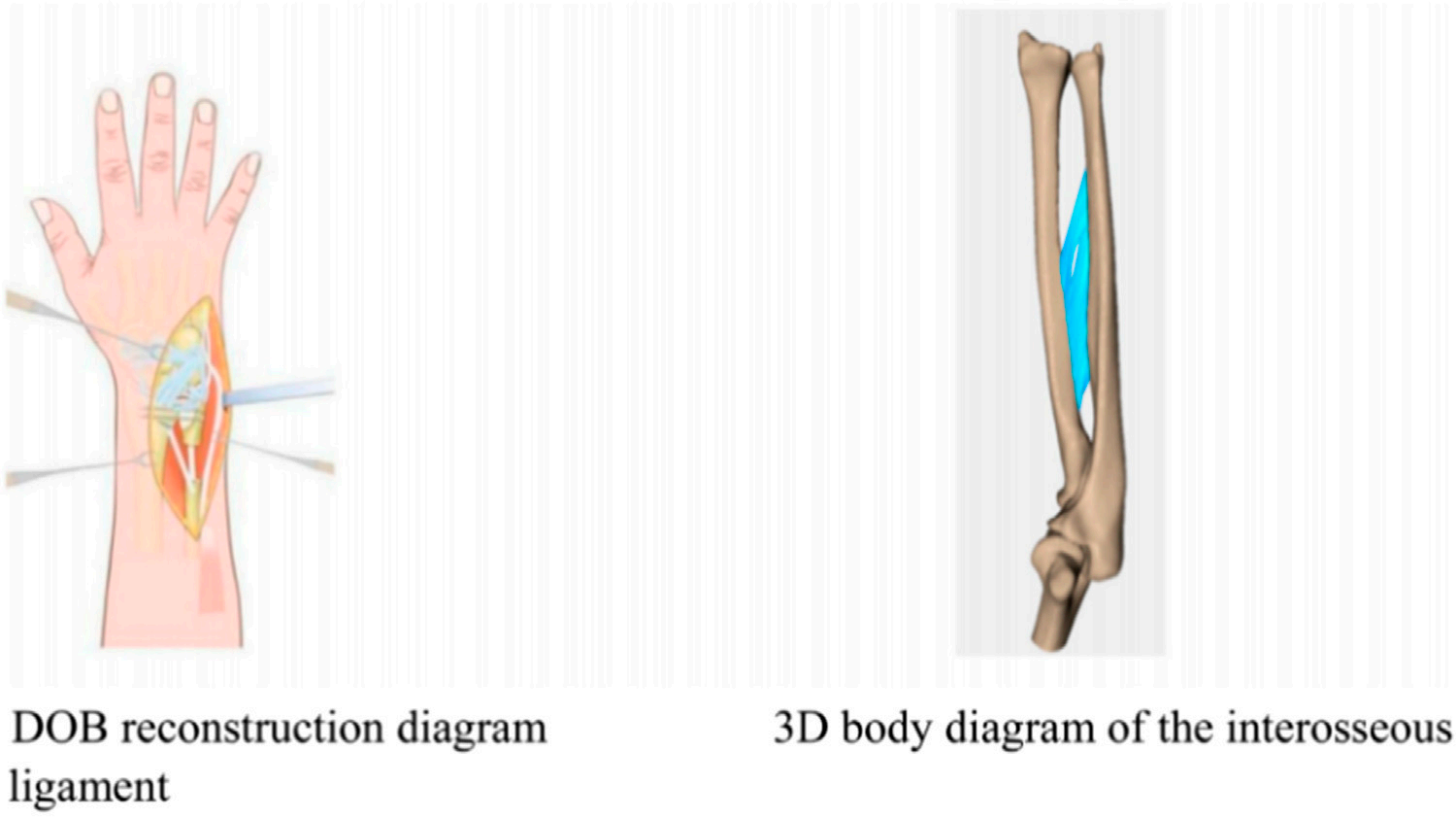
DOB reconstruction and schematic diagram of the interosseous wrist ligament.

On the basis of the reverse-engineered wris joint STP model and by utilizing the anatomical relationships derived from 3Dbody software, the model included the articulating surfaces of the joint cartilage between bones, interosseous membrane ligaments, extensor tendons (such as the DOB tendon), and elbow joint ligaments (Figure 2). The assembly completed the wrist joint STP solid geometric model. The specifications included a 3 mm diameter for the DOB tendon, a 20 mm height for ulnar excision, and two screws with diameters of 3.5 mm.

**FIGURE 3:**
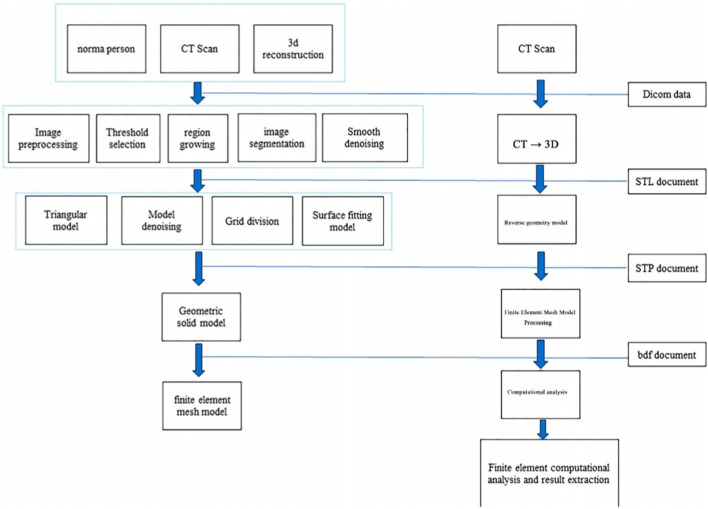
Schematic diagram of three-dimensional CT reconstruction steps and the technical process.

**FIGURE 4:**
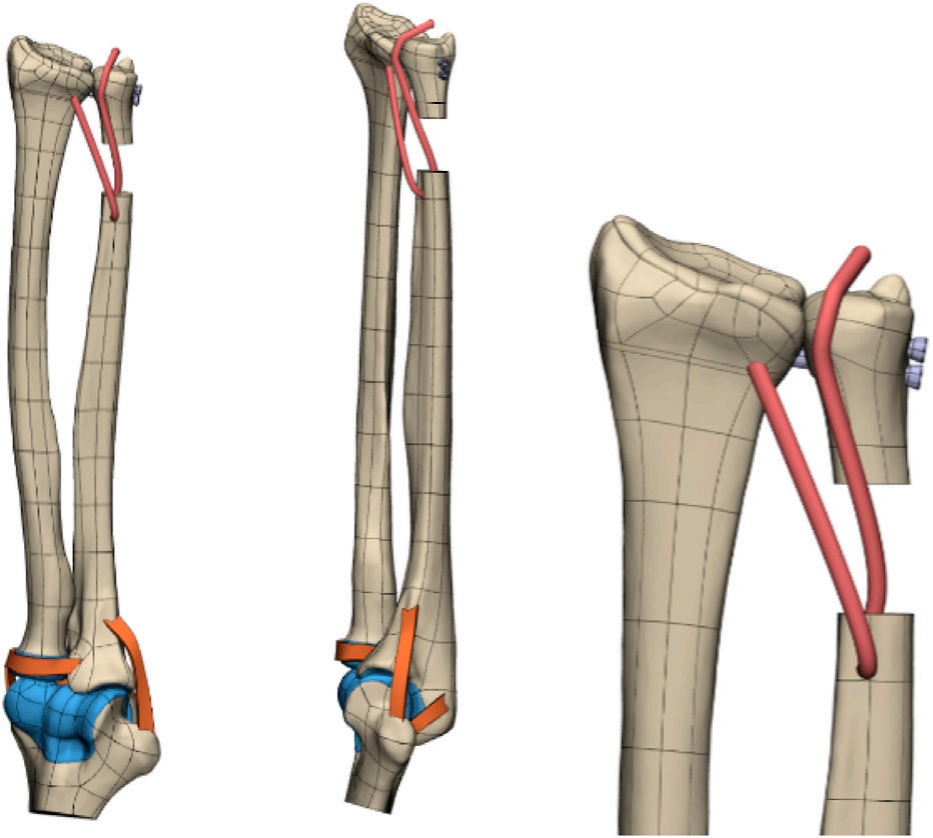
Reverse drawing of the 3D wrist joint model (DOB reconstruction).

**FIGURE 5:**
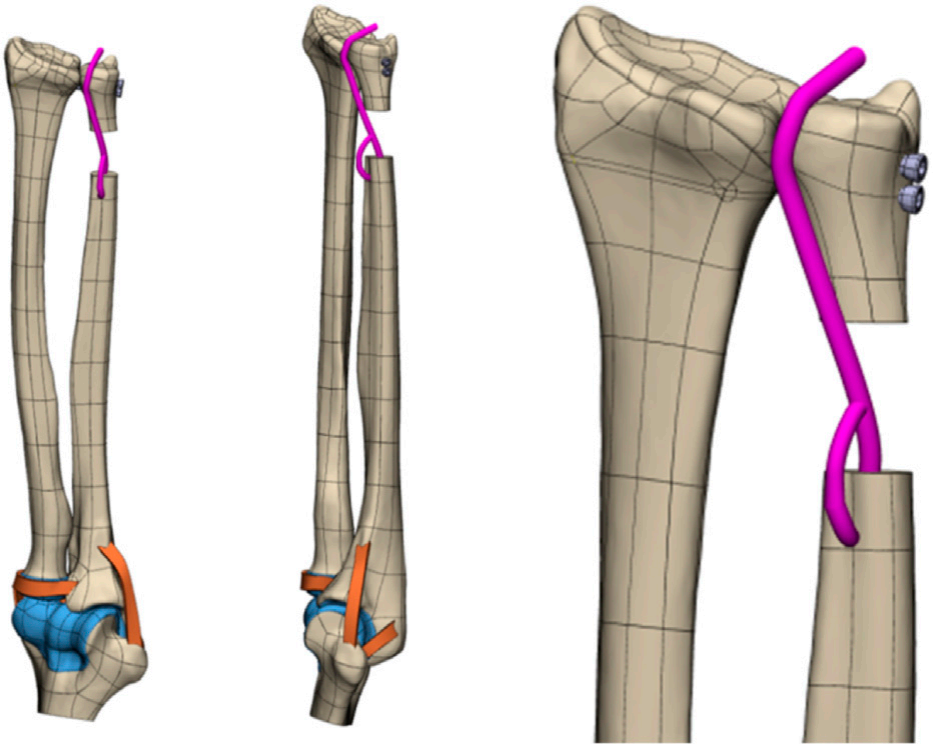
Reverse drawing of the 3D wrist joint model (without DOB reconstruction).

The 3D CT reconstruction steps, technical processes (Figure 3), and reverse engineering scheme of the model are illustrated in the following figures (Figures 4, 5).

#### 2.1.3 Finite-element geometric model refinement stage

**FIGURE 6:**
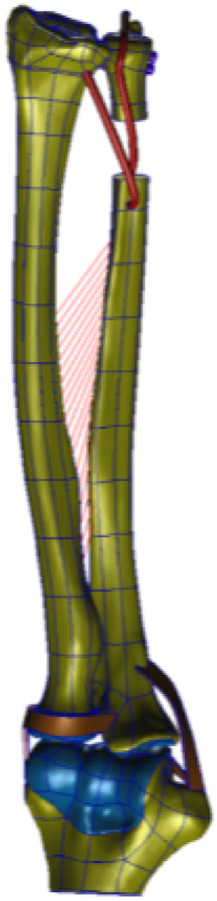
(DOB reconstruction) Finite-element geometric model diagram of the DOB tendon reconstruction process for the wrist joint.

**FIGURE 7:**
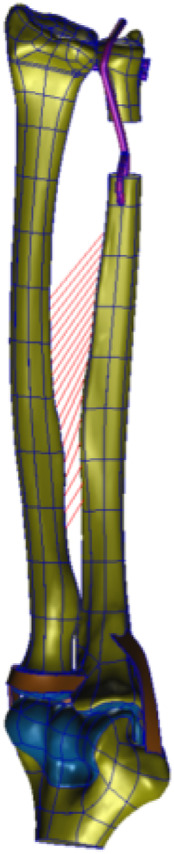
(Without DOB reconstruction) Finite-element geometric model diagram of the DOB tendon reconstruction process for the wrist joint.

The wrist joint model, refined through reverse engineering, was supplemented with the main ligaments of the elbow and wrist joints, including the DOB tendon. Watanabe et al. (Watanabe et al., 2005; Kitamura Takashi et al., 2011) emphasized the importance of the distal interosseous membrane ligament, which limits the palmar and dorsal instability of the distal radioulnar joint in all forearm rotation positions. As a crucial linkage unit between the distal radius and ulna, the distal interosseous membrane cannot be overlooked. Ligaments near the distal joint of the wrist were not considered; thus, this study conducted a biomechanical finite-element analysis of the stability of the proximal ulnar remnants after performing DOB tendon reconstruction while considering the role of the distal interosseous membrane between the radius and ulna. The wrist joint ligaments were simulated via 1D spring elements to implement an equivalent modeling procedure. The refined geometric wrist joint model, reversed and optimized, was imported into the finite-element preprocessing software MSC. Patran 2019 (NASA Company, America), as illustrated in (Figures 6, 7).

### 2.2 Finite-element mesh division

The STP file containing the geometric model of the DOB in the wrist joint was imported into finite-element preprocessing software MSC. Patran 2019 (NASA Company, America) for setting the properties of the finite-element mesh, defining material parameters, applying loads, setting boundary condition constraints, and preparing for the subsequent finite-element analysis step.

**FIGURE 8:**
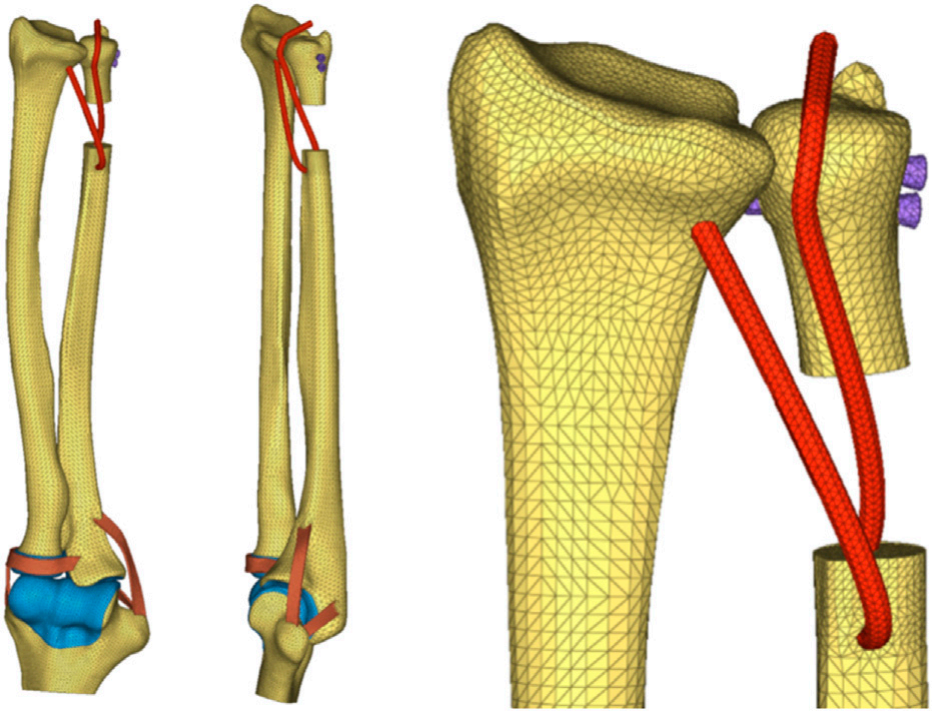
(DOB reconstruction) Mesh division of the DOB of the wrist joint in HyperMesh software.

**FIGURE 9:**
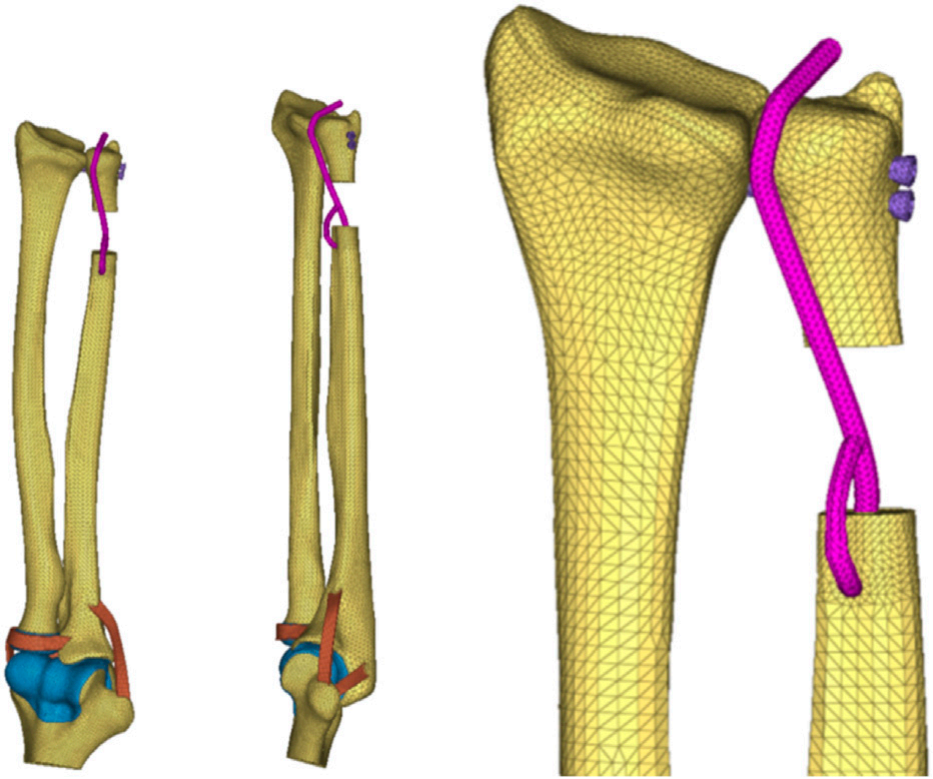
(Without DOB reconstruction) Mesh division of the wrist tendon in HyperMesh software.

**FIGURE 10:**
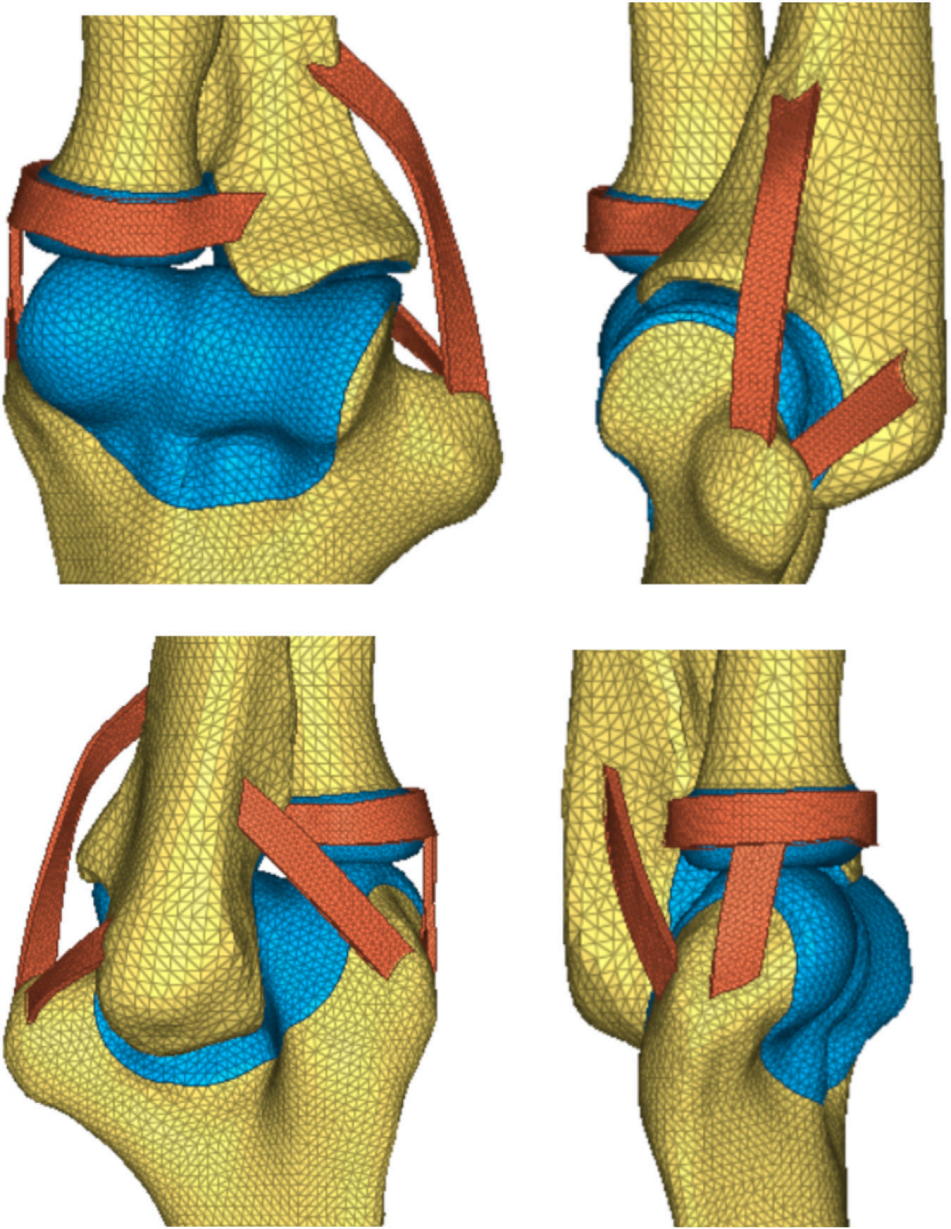
Zoom in a detail of the ligaments model.

After preprocessing, the models were submitted to the finite-element postprocessing software, MSC. Nastran 2019 (NASA Company, America), for computational analysis and results visualization purposes. Various groups of finite-element mesh models are illustrated in (Figures 8–10).

Finite-Element Mesh Division: The reconstructed DOB group comprised a total of 36,714 nodes and 160,400 elements; the nonreconstructed DOB group comprised 36,164 nodes and 159,136 elements. The skeletal structures, DOB tendons, articular cartilage, and screws adopted TetMesh Tet4 tetrahedral solid mesh elements. One-dimensional spring elements were used for an equivalent simulation of the interosseous ligaments.

### 2.3 Material parameter settings

**FIGURE 11:**
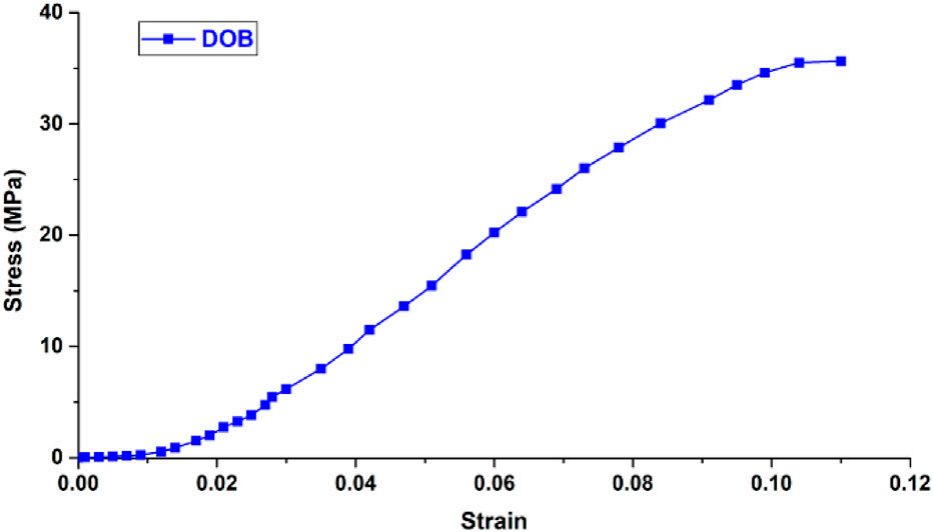
Stress-strain curve of the DOB tendon in the wrist joint.

**FIGURE 12:**
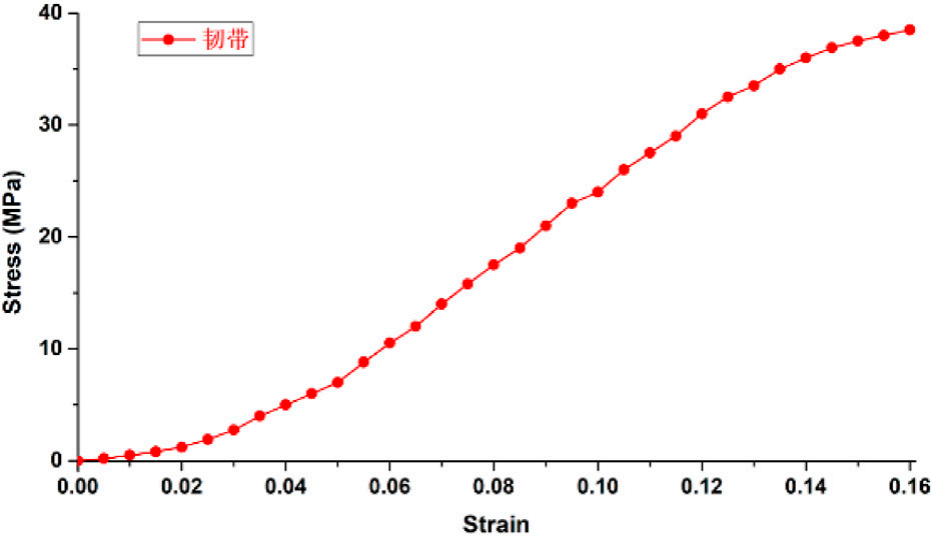
Ligament stress‒strain curve.

The wrist joint bones, articular cartilage, and screws were assumed to be isotropic, homogeneous, and continuous linear elastic materials. The radioulnar joint distal radioulnar ligament was modeled as a linear elastic spring ligament (Guo, 2007) with a stiffness coefficient of 50 N/mm. Ligaments and wrist joint DOB tendons (Abramowitch et al., 2010; Mesfar and Shirazi-Adl, 2005; Mesfar and Shirazi-Adl, 2006) were set according to the material parameters of human joint tendon or ligament reconstruction surgery and were assumed to be nonlinear materials subjected to tension only. The friction coefficient between the DOB tendons and the hole at the distal end of the radius was set to 0.3 (Wan et al., 2017), whereas the friction coefficient between the articular cartilage and the triangular fibrocartilage complex was 0.001 (Zhang, 2009). The other material parameters are shown in the Table 1 and Figures 11, 12.

**TABLE 1:** Wrist joint material parameters.

	Elastic modulus (MPa)	Poisson’s ratio
Bones (Takatori et al., 2002)	15,000	0.30
Articular cartilage (Renani et al., 2017; Brown et al., 2009)	0.7	0.47
Ligament (Yan, 2019)	500	0.48
DOB tendon (Abramowitch et al., 2010; Mesfar and Shirazi-Adl, 2005; Mesfar and Shirazi-Adl, 2006)	215.3	0.40
Screw (Ti-6Al-4V) (Verim et al., 2014)	106,000	0.33

### 2.4 Boundary condition assumptions

**FIGURE 13:**
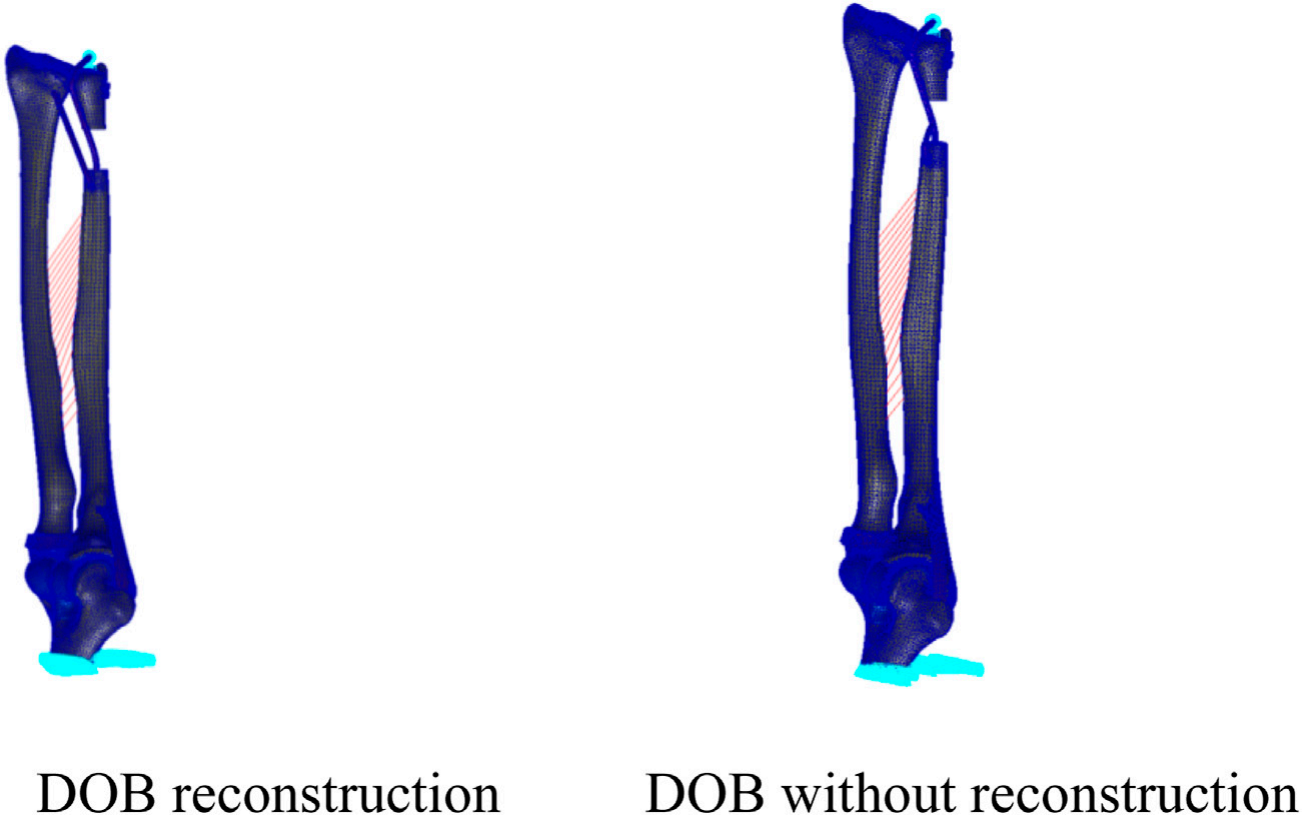
Boundary conditions of the finite-element DOB model for the wrist joint.

For the finite-element model of the DOB in the wrist join, loading and boundary constraints were applied by following the approach of Kitamura et al., (2011a): the proximal ulna was fixed, and a push or pull load of 20 N was applied on the palmar or dorsal side of the distal radius, resulting in three different wrist joint motion states (neutral, 60° pronation, and 60° supination), as shown in Figure 13.

### 2.5 Biomechanical finite-element analysis of the DOB in the wrist joint

**FIGURE 14:**
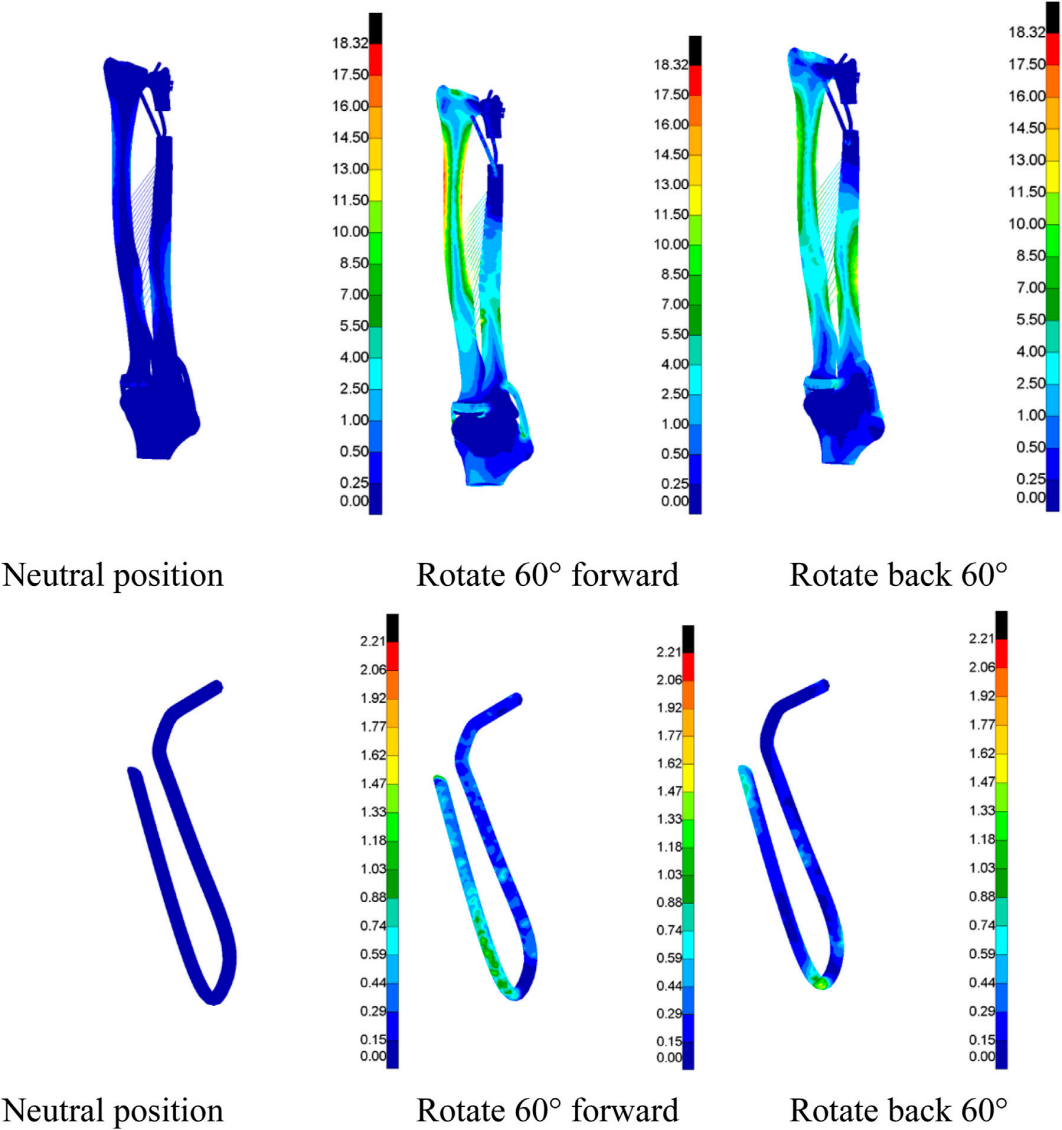
(DOB reconstruction) Von Mises stress cloud map of the DOB of the wrist joint (MPa).

**FIGURE 15:**
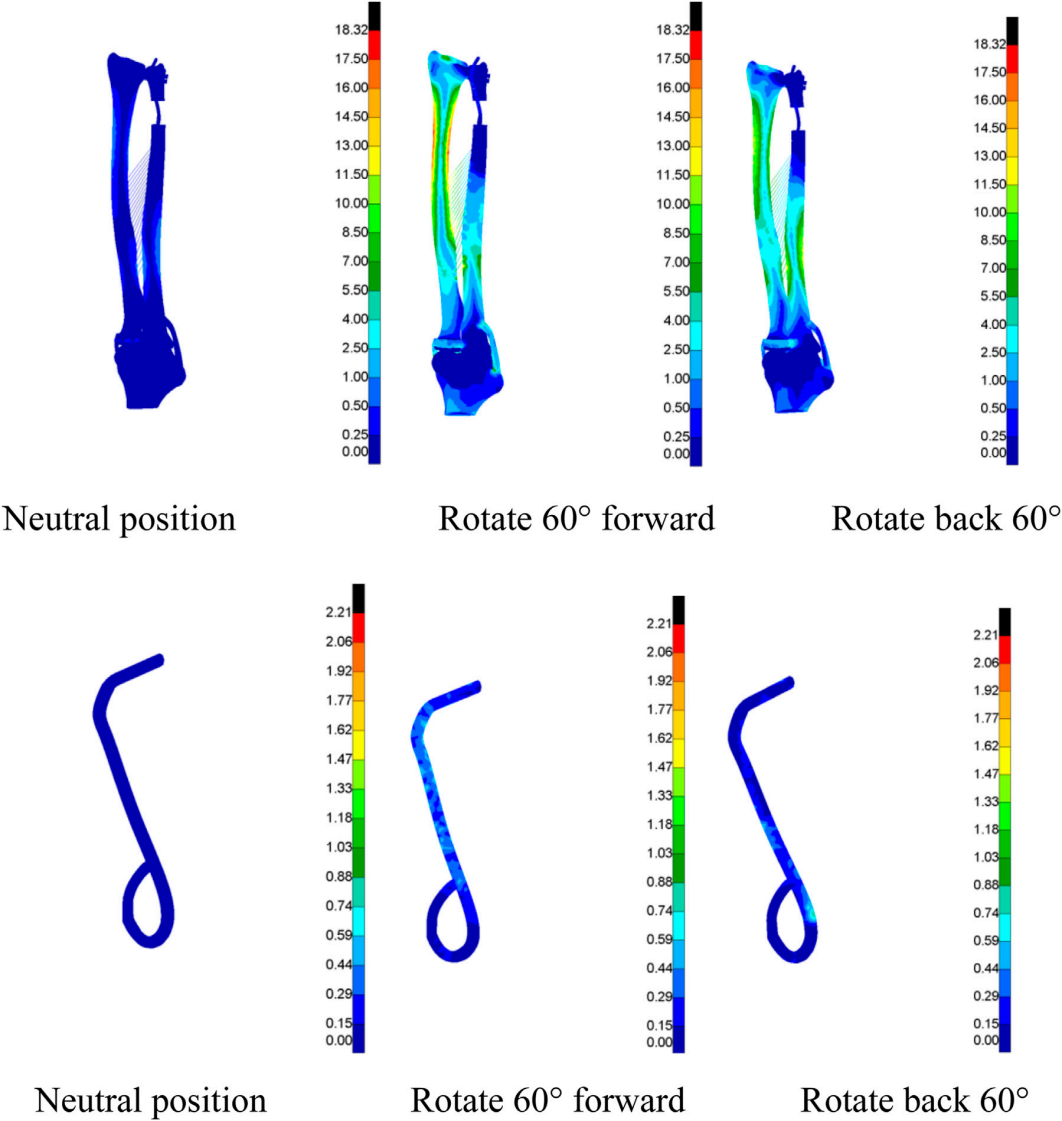
(Without DOB reconstruction) Von Mises stress cloud map (MPa) of the tendon.

A finite-element simulation analysis was conducted on the DOB tendons of the wrist joint to obtain Von Mises stress cloud maps under three different motion states (neutral, 60° pronation, and 60° supination) to more clearly and intuitively observe their distribution characteristics. Each group was uniformly scaled for a specific data analysis (Figures 14, 15).

### 2.6 Data analysis

Through a finite-element simulation analysis of DOB tendons in the wrist joint, data on the displacement from the distal end of the radius to the ulna and the Von Mises stress of the bones and DOB tendons were obtained under three different motion states (neutral, 60° pronation, and 60° supination).

## 3 Results

Tables 2, 3 present the data acquired regarding the displacement of the distal radius relative to the ulna and the Von Mises stress in the bones and DOB tendons of the wrist joint under three different motion states (neutral, 60° pronation, and 60° supination); the reconstructed and nonreconstructed DOB conditions were compared.

**TABLE 2:** (DOB reconstruction) Displacement and structural stress intensity of the radius relative to the proximal residual end of the ulna.

	Neutral position	Rotate 60° forward	Rotate back 60°
Displacement of the radius relative to the proximal residual end of the ulna (mm)	17.89	12.57	15.53
Bone stress (MPa)	1.01	18.32	14.69
DOB tendon stress (MPa)	0.07	2.21	1.55

**TABLE 3:** (DOB without reconstruction) Displacement and structural stress intensity of the radius relative to the proximal residual end of the ulna.

	Neutral position	Rotate 60° forward	Rotate back 60°
Displacement of the radius relative to the proximal residual end of the ulna (mm)	18.05	14.62	16.89
Bone stress (MPa)	1.02	18.29	14.41
DOB tendon stress (MPa)	0.03	0.87	0.85

### 3.1 Structural stress

#### 3.1.1 Comparative analysis

**FIGURE 16:**
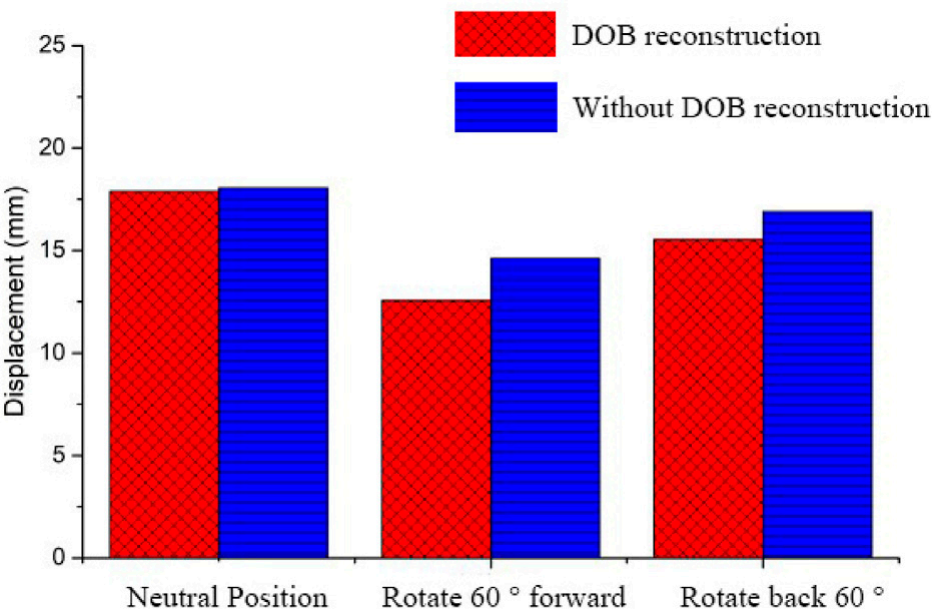
Comparison regarding the displacement of the radius relative to an ulna fracture.

**FIGURE 17:**
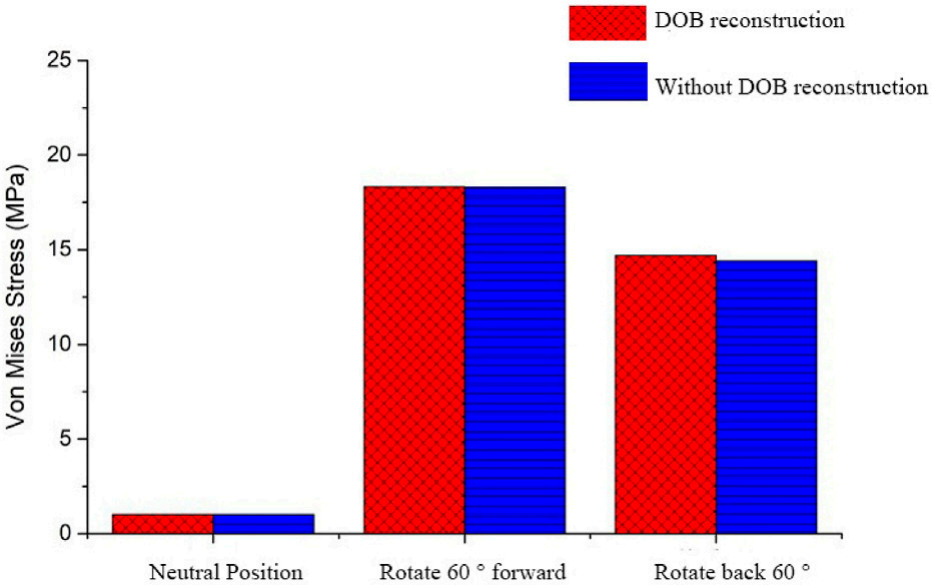
Comparison between the peak equivalent stress values of the radial and ulnar bones.

**FIGURE 18:**
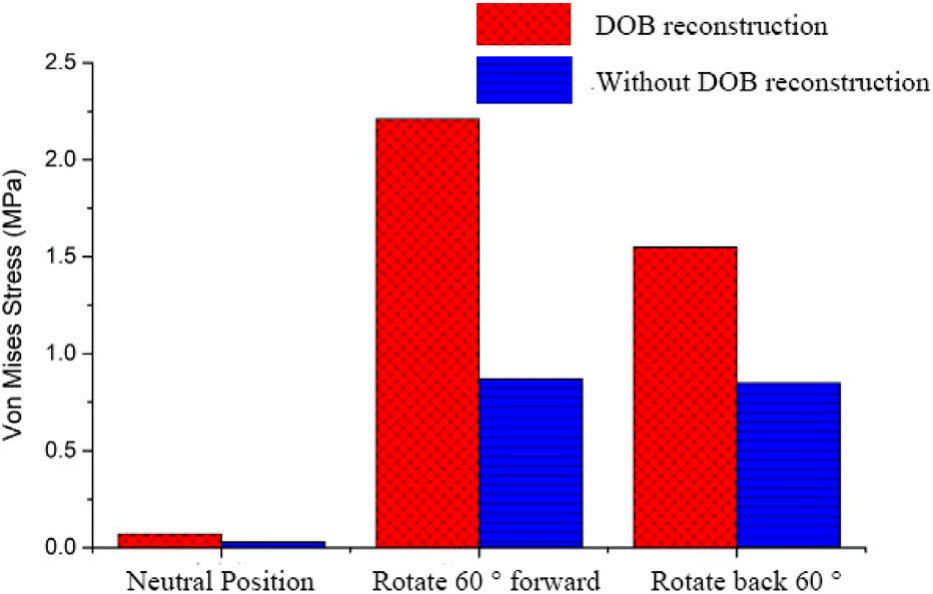
Comparison between the equivalent stress peak values representing DOB muscle health.

In DOB reconstruction surgery, the displacement of the distal radius relative to the proximal ulnar remnants varied as follows: approximately 17.89 mm in the neutral position, 12.57 mm at 60° pronation, and 15.53 mm at 60° supination (Figure 16). Additionally, from the stress analysis of the ulnar remnants and DOB tendons, the peak bone stresses at the proximal ulnar remnants were 1.01 MPa in the neutral position, 18.32 MPa at 60° pronation, and 14.69 MPa at 60° supination (Figure 17). The peak stresses within the DOB tendons were 0.07 MPa in the neutral position, 2.21 MPa at 60° pronation, and 1.55 MPa at 60° supination (Figure 18).

In surgeries without DOB reconstruction, the displacement of the distal radius relative to the proximal ulnar remnants measured approximately 18.05 mm in the neutral position, 14.62 mm at 60° pronation, and 16.89 mm at 60° supination (Figure 16). The peak bone stresses at the proximal ulnar remnants were 1.02 MPa in the neutral position, 18.29 MPa at 60° pronation, and 14.41 MPa at 60° supination (Figure 17). The peak stresses within the DOB tendons were 0.03 MPa at the neutral position, 0.87 MPa at 60° pronation, and 0.85 MPa at 60° supination (Figure 18).

## 4 Discussion

Sauve-Kapandji (S-K) surgery is a salvage operation for distal radioulnar joint (DRUJ) arthritis, but it often leads to postoperative instability of the proximal ulna and clinical postoperative pain and discomfort in the wrist. At present, the proximal stump stabilization technique of ulna is often used.

Kitamura et al. (2011b) proposed the hypothesis that DOB reconstruction after Sauve-Kapandji surgery has a role in the stability of the proximal ulnar stump, but no experiments have proved this claim. In addition, when the stability of the ulnar stump needs to be reconstructed after S-K surgery, the reconstruction of graft stress and the position of the forearm are the key to successful results.

Utilizing the finite-element method (FEM), biomechanical simulations were conducted to determine whether performing distal oblique bundle (DOB) reconstruction during the Sauvé-Kapandji (S-K) procedure provides stability to the proximal ulna during three different motion states (neutral, 60° pronation, and 60° supination). The analysis focused on the displacement changes in the distal radius relative to the ulna, as well as the stress variations exhibited by the bones and DOB tendons.

When DOB reconstruction was performed during the S-K procedure, different motion states resulted in varied displacements of the distal radius relative to the ulna toward the palmar or dorsal side with the neutral position having the highest displacement at approximately 17.89 mm, followed by supination at approximately 15.53 mm and pronation at approximately 12.57 mm,without DOB reconstruction showed obvious displacement with the neutral position having the highest displacement at approximately 18.05 mm; followed by supination at approximately 16.89 mm and pronation at approximately16.89 mm,The increase of displacement was 0.89%, 16.31% and 8.76%, respectively. The increase of displacement corresponding to neutral position was not obvious, and the most significant increase was at 60° pronation, followed by 60° supination. It can be shown that DOB tendon reconstruction prevented significant collisions between the radius and ulnar remnants,. After DOB tendon reconstruction, the displacement of the volar or dorsal radioulnar joint in pronation and supination was limited, especially in supination position. Therefore, with the help of biomechanical theory, it is found that DOB tendon reconstruction surgery plays a positive role in the stability of the proximal ulna stump.

The displacement values showed a trend of neutral > supination > pronation, the reason is that the highest stress at the forearm interosseous membrane in the neutral position of the forearm decreases with the increase of pronation and supination (Harrison et al., 2005). In addition, radiological and anatomical studies of the changes in the interosseous distance between the radius and ulna during forearm rotation in cadaveric subjects have also found that the neutral position provides the widest interosseous distance (Farr et al., 2015),Therefore, DOB reconstruction should be fixed at the minimum tension in supination position, which is basically consistent with the clinical findings.

Moreover, a stress analysis of DOB reconstruction tendons revealed varying stress patterns across the different motion states. The peak stresses in the DOB tendons were 0.07 MPa in the neutral state, 2.21 MPa in the pronation state, and 1.55 MPa in the supination state. Compared with the other motion states, pronation resulted in the highest stresses in tendons, highlighting the biomechanical differences induced by varying degrees of motion. Therefore, in clinical DOB reconstruction, the graft tension should be maximized, and the graft should be tensioned at 60° pronation when the distance between radius and ulna is the shortest. However, postoperative plaster fixation of the forearm should be performed at 60° supination tension for about 6 weeks to obtain optimal graft firmness. Furthermore, during postoperative rehabilitation or daily activities, such as holding an object at forearm level in a neutral or pronation position, with load applied to the volar side and forearm in pronation position, the proximal ulna stump moves to contact the extensor carpal ulnaris tendon. Even maintaining a 1 kg load at such a forearm position may result in tendon irritation. Therefore, guidance in postoperative rehabilitation and daily activities should be considered, including instructing the patient to hold the object in a supination position with the forearm whenever possible, and active pronation of the forearm should be prevented by 60°.

However, the peak bone stresses were comparable between the groups, with minimal differences observed. Notably, the tendon stress in the pronation position decreased without DOB reconstruction, suggesting a reduced postoperative tendon load capacity.

This study utilized CT imaging reconstruction and reverse engineering techniques to create a three-dimensional model of the wrist joint and DOB tendons. An FEM analysis provided quantitative and qualitative biomechanical insights into the role of DOB tendon reconstruction in terms of stabilizing the proximal ulnar remnants under different wrist joint motion states. These findings contribute valuable biomechanical principles and structural engineering insights, offering guidance for future clinical studies on wrist joint surgeries.

## Data Availability

The original contributions presented in the study are included in the article/supplementary material, further inquiries can be directed to the corresponding author.
